# Convertible Thermal Meta-Structures via Hybrid Manufacturing of Stereolithography Apparatus 3D Printing and Surface Metallization for Thermal Flow Manipulation

**DOI:** 10.3390/polym15010174

**Published:** 2022-12-29

**Authors:** Bo Li, Jianrui Zhang, Tianxiang Deng, Facai Ren

**Affiliations:** 1 School of Mechanical and Power Engineering, East China University of Science and Technology, Shanghai 200237, China; 2 Additive Manufacturing and Intelligent Equipment Research Institute, East China University of Science and Technology, Shanghai 200237, China; 3 Shanghai Collaborative Innovation Center for High-End Equipment Reliability, Shanghai 200237, China; 4 Shanghai Institute of Special Equipment Inspection and Technical Research, Shanghai 200062, China

**Keywords:** thermal metamaterial, 3D printing, thermal manipulation, thermal cloak, thermal concentration

## Abstract

For manipulating heat flow according to human desire, thermal metamaterial structures (meta-structures) have attracted growing interest. Based on the transformation thermotics and the effective medium approximation theory, we designed and fabricated a convertible thermal meta-structural device to demonstrate that multiple different heat-flow manipulations could be conducted using a single thermal meta-structural device. The convertible meta-structures were designed by discretizing a two-dimensional plane and separating multiple square unit modules with stripe-shaped dissimilar materials of the Polydimethylsiloxane (PDMS) and solid resin with surface metallized copper (Cu). The convertible thermal meta-structure device with a relatively high geometric accuracy was fabricated via a proposed hybrid manufacturing path of “Stereolithography Apparatus (SLA) 3D printing—electroless plating—electroplating—thermally insulated packaging”. The thermal manipulation features were numerically simulated and preliminarily verified by experimental testing. Using multiple dispersed square unit modules to replace an annular region of the thermal meta-structure exhibited different thermal flow functions, including thermal cloak, thermal concentration, thermal rotation, and thermal dispersion, through the unique geometric design of the heat-flow transfer direction within each module. By rotating each square unit module at a specific angle and arranging the modules, similar to a “jigsaw puzzle”, the convertibility among different thermal manipulation functions was achieved. This path is anticipated to provide a new strategy for multifunctional meta-structures in thermo-physics and its potential engineering applications.

## 1. Introduction

Manipulating thermal flow according to human desire is not always easy. According to Fourier’s law, thermal energy diffuses from high to low temperatures without an external source. Nonetheless, many recent developments in metamaterial technology in information science have contributed to several innovative advances in thermal flow manipulation. Metamaterial, also called metamaterial structure (meta-structure), is an artificially designed structure with unique material characteristics or bizarre physical properties that are difficult or impossible to discover in nature [1,2,3]. Meta-structures have innovative functionalities as a result of ingenious artificial design, creating various physics devices, like invisible cloaks [4], carpets [5], invisible sensors [6], illusion devices [7,8,9] or hyperlenses [10,11]. Coordinate transformation idea is the design basis of meta-structures [1]. Transformation optics was first proposed to perform cloaking on electromagnetic waves, based on form-invariance of governing equations after coordinates transformation [1]. Since then, the concept was first extended to the thermal fields [12]. The transformation thermotics, as a counterpart of transformation optics, has been proposed to guide heat flux in thermal manipulation and generate some novel thermal meta-structural devices, such as heat cloaks [13,14,15,16,17], thermal energy harvesting devices [18], and thermal sensors [6]. Thermal meta-structures have attracted significant interest because they offer great flexibility to tune heat flow for desired thermal functionalities [19,20]. Nowadays, the thermal manipulation concept has been extended to incorporate the macroscopic thermal diodes with switching functions [21,22], the manipulation of thermal waves [23], and the complete control and convertible manipulation of multiple heat signatures [24,25,26].

It is even more challenging for one thermal meta-structural device to exhibit more than two functions of thermal flow manipulation. That is one of the main interests of this work. Generally, once the configuration and constituent materials of a thermal meta-structural device are designed, the corresponding functionality can only be achieved at certain conditions [27,28,29]. Most thermal metamaterials are usually unable to adapt dynamically to the change of environment. Several researchers have begun to notice this issue and make new attempts to implement multiple different thermal manipulations and other thermal functions on a single thermal meta-structural device [17,24,25,26]. For instance, it can be achieved by flexibly controlling the switch from partial concentration to uniform concentration and from rotation to the concentration of heat flow [25]. A concept of adaptive thermal manipulations to exhibit more distinct thermal behaviors, such as thermal cloaking, concentrating, and rotation, by one meta-structural device under different heat-flow directions has been promoted recently [17,25,26]. This path is anticipated to provide a new strategy for multifunctional metamaterial design in the thermal field.

Furthermore, the thermal meta-structures, designed according to transformation thermotics, commonly present challenges in fabricating these complex structures and estimating their properties. To artificially build thermal meta-structural devices, the requisite metamaterial properties are achieved by specifically engineered materials with inherent inhomogeneities and/or anisotropies. To overcome these manufacturing challenges, additive manufacturing (AM) techniques, also popularly called 3D printing, including laser powder bed fusion (L-PBF), stereo lithography appearance (SLA), and two-photon polymerization (TPP) micro-AM, can provide new feasible routes for the fabrication of thermal meta-structural devices. Although there are still not many reports on fabricating thermal metamaterials by 3D printing, our previous work has verified the feasibility of L-PBF, also called selective laser melting (SLM), to build metal-contained thermal meta-structures of thermal concentration and thermal rotation [30]. Nonetheless, when it comes to 3D-printed high-precision metal structures, the L-PBF barely struggle to meet the precision fabrication needs of miniature structures. It is due to that the L-PBF remains challenging on as-printed good surface roughness, especially for the overhanging structures [31,32]. In contrast, the SLA-printed structures have better surface roughness and geometric accuracy [33]. However, the SLA cannot directly print metal material structures.

Hence, this work proposed a hybrid plating path to achieve surface metallization on SLA-printed resin high-precision structures using the electroless plating—electroplating technique route. Here we called the method of SLA combined with hybrid plating a “hybrid manufacturing” method for metal Cu-contained thermal meta-structural devices. In this work, we designed and fabricated a convertible thermal meta-structural device to demonstrate the concept that a single thermal meta-structural device could perform different heat-flow manipulations. The so-called “convertible” means that various thermal properties of the meta-structure can be converted to each other through the ordered matching of modular components in the meta-structural device. This device exhibited four thermal manipulation functions: thermal concentration, cloak, rotation, and dispersion. We used polydimethylsiloxane (PDMS) and surface metallized copper on the SLA-printed resin to form the thermal meta-structural device. The simulation of thermal flow manipulation on the convertible thermal meta-structures was conducted using COMSOL Multiphysics Software. Then, the thermal-flow manipulating functions of the convertible thermal meta-structural device were experimentally verified through a self-built thermal test platform. The work sheds new light on the design and engineered facile fabrication of convertible thermal meta-structures. It is beneficial to expand the possible application scope of multifunctional thermal metamaterials.

## 2. Fundamental Design Theory of Thermal Meta-Structures

### 2.1. The Transformation Thermotics Theory

Thermal conduction, thermal convection, and thermal radiation are three modes of thermal energy transfer. The physical form of thermal radiation is microwave, which naturally conforms to the theory of transformation optics. The physical equations of thermal convection are relatively complex, with weak effects on solids. Hence, only the heat conduction process is considered in the transformation thermotics theory [12,25].

According to Fourier’s law, at any point, denoted as *x* = (*x*, *y*), in a two-dimensional space, denoted as *Ω*, the heat conduction equation without an internal heat source is:

(1) $$\rho (x)c(x)\frac{\partial T}{\partial t}-\nabla \cdot (\kappa (x)\cdot \nabla T)=0$$

where *ρ* is the mass density of the material, *c* is the material’s specific heat capacity, *T* is the temperature, *t* is the time, and *k* is the material’s thermal conductivity. The basic idea of transformation optics theory is coordinate transformation [1]. During the coordinate transformation process, the Maxwell equation set has the form-invariance, meaning that only the material parameters and physics-field variables will change. Thus, manipulating electromagnetic wave propagation can be achieved by controlling the material parameters. Since the fundamental physics laws are applicable in any coordinate space, the characteristic physical flux of any physical field can be propagated along the tailored route by distorting the coordinate space. Similar to the transformation of Maxwell equations, the thermal conduction equation also has form-invariance during the process of coordinate transformation, resulting in only the relevant parameters of the material and the thermal field can change [34].

For coordinate transformation, denoted as *x* = (*x*, *y*) → *x*′ = (*x*′, *y*′), from the coordinate space, *Ω*, to coordinate space, *Ω*′, Jacobian matrix, denoted as **J** = ∂(*x*′, *y*′)/∂(*x*, *y*), can be used to describe it. The thermal conduction equation of the space, *Ω*′, after coordinate transformation is [34]:

(2) $$\rho (x^{\prime })c(x^{\prime })\det(J)\frac{\partial T^{\prime }}{\partial t}-\nabla \cdot (J^{-T}\kappa (x^{\prime })J^{-1}\det(J)\nabla T)=0$$

Since Equations (1) and (2) have the form-invariance, the thermal conductivity parameters in the space, *Ω*′, after coordinate transformation can be obtained as:

(3) $$k^{\prime }=J^{-T}kJ^{-1}\det(J)=kJ^{-T}J^{-1}\det(J)=kT^{-1}=\frac{JkJ^{T}}{\det(J)}$$

where *k* is the thermal conductivity of the original coordinate space denoted as *Ω*, *k*′ is the thermal conductivity of the transformed coordinate space denoted as *Ω*′, and **T** is the coordinate transformation matrix.

**Figure 1:**
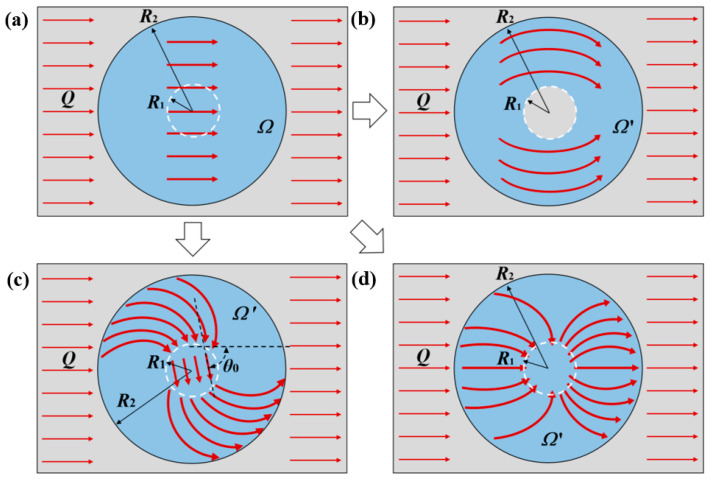
Schematic diagrams of coordinate transformations from an original plane space (**a**), *Ω*, to a plane space, *Ω*′, of thermal cloak meta-structure (**b**), thermal rotation meta-structure (**c**), or thermal concentration meta-structure (**d**).

According to the theory of transformation thermotics, the thermal conductivity of the space, *Ω*′, after coordinate transformation is related to the transformation matrix of coordinate transformation, **T**. Thus, the space thermal conductivity is determined by the mode or route of coordinate transformation. Therefore, the critical point of the structural design of the thermal metamaterials lies in the coordinate transformation mode. Since different coordinate transformation modes can produce different thermal meta-structural functions, the coordinate transformations of thermal cloak, thermal concentration, and thermal rotation meta-structures were conducted as follows, respectively, as illustrated in Figure 1. While for the thermal dispersion meta-structure, its coordinate transformation mode is similar to that of the thermal concentration meta-structure.

### 2.2. Application of the Transformation Thermotics on Thermal Cloak Meta-Structure

Figure 1a,b depict the plane circular structure spaces before and after the coordinate transformation for designing a thermal cloak meta-structure. A circular space with a radius marked as *R*_1_ in the center of the annular space marked as *Ω*′ is the thermal cloaking space. Before the coordinate transformation, the thermal flow, denoted as *Q*, is transmitted along the horizontal straight lines inside and outside the thermal cloak space (Figure 1a). After the coordinate transformation, this space is transformed into two parts (Figure 1b). Although the heat flows, *Q*, are still transmitted in the horizontal straight lines outside the annular space, *Ω*′, after it enters the *Ω*′ space, it bypasses the central circular area with a radius of *R*_1_, and transmits annularly forward along the annular area (Figure 1b). In other words, the temperature gradient is zero in the central circular area as the thermal cloak space, so the external thermal-flow transfer has no effect. That can be achieved by a coordinate transformation that compresses a circular region into an annular region. Its coordinate transformation mode can be stated as follows.

(4) $$\{\begin{array}{c} r^{\prime }=R_{1}+\frac{\left(R_{2}-R_{1}\right)}{R_{2}}r\\ \theta^{\prime }=\theta \end{array}$$

Accordingly, the Jacobian matrix computing the coordinate transformation denoted as (*x*, *y*) → (*r*, *θ*) → (*r*′, *θ*′) → (*x*′, *y*′) is:

(5) $$J_{{xx}^{\prime }}=J_{xr}\cdot J_{{rr}^{\prime }}\cdot J_{r^{\prime }}{}_{x^{\prime }}=R(\theta )\mathrm{diag}(\alpha^{-1},\;r/r^{\prime })R(\theta^{\prime })$$

where *R*(*θ*) and *R*(*θ*′) are the rotation matrix, and *α*, **J***_xr_*, **J***_rr_*_′_, and **J***_r_*_′*x*′_ can be denoted as:

(6) $$\alpha =[R_{2}(\theta )-R_{1}(\theta )]/R_{2}(\theta )$$

(7) $$J_{xr}=\partial (x,y)/\partial (r,\theta )$$

(8) $$J_{rr^{\prime }}=\partial (r,\theta )/\partial (r^{\prime },\theta^{\prime })$$

(9) $$J_{r^{\prime }x^{\prime }}=\partial (r^{\prime },\theta^{\prime })/\partial (x^{\prime },y^{\prime })$$

Bringing the above parameters and the denoted det(**J***_xx_*_′_) = *α*^−1^ *r/r*′ into Equation (3), the coordinate transformation matrix is obtained as:

(10) $$T^{-1}=J_{xx^{\prime }}^{-T}J_{xx^{\prime }}^{-1}\det(J_{xx^{\prime }})=R(\theta^{\prime })\mathrm{diag}(\alpha r/r^{\prime },\alpha^{-1}r^{\prime }/r)R{\left(\theta^{\prime }\right)}^{-1}$$

Since *R*(*θ*)^−1^ = *R*(*θ*)^T^, and *θ*′ = *θ*, according to Equations (3) and (10), the thermal conductivity of the annular space, *Ω*′, after coordinate transformation is calculated as:

(11) $$k^{\prime }=kT^{-1}=R(\theta \prime )\mathrm{diag}(k_{r}^{\prime },k_{\theta }^{\prime })R{\left(\theta {}^{\prime }\right)}^{T}$$

After simplifying Equation (11), thermal parameters in the annular space, *Ω*′, of the thermal cloak meta-structure after coordinate transformation can be obtained as:

(12) $$\{\begin{array}{c} {k^{\prime }}_{r}=\frac{r^{\prime }-R_{1}}{r^{\prime }}\\ {k^{\prime }}_{\theta }=\frac{r^{\prime }}{r^{\prime }-R_{1}} \end{array},\;\rho^{\prime }c^{\prime }=\frac{r^{\prime }-R_{1}}{r}{\left(\frac{R_{1}}{R_{2}-R_{1}}\right)}^{2}\left(\rho c\right)$$

Dividing the coordinate-transformed parameters in Equation (12) by det(**J***_xx_*_′_), to simplify the material parameters, the thermal parameters can be further obtained as:

(13) $$\{\begin{array}{c} k_{r}^{\prime \prime }={\left(\frac{R_{2}}{R_{2}-R_{1}}\right)}^{2}{\left(\frac{r^{\prime }-R_{1}}{r^{\prime }}\right)}^{2}k_{0}\\ k_{\theta }^{\prime \prime }={\left(\frac{R_{2}}{R_{2}-R_{1}}\right)}^{2}k_{0} \end{array},\;\rho^{\prime \prime }c^{\prime \prime }=\rho c$$

where *ρc* is the specific heat per unit volume, thus, it can be inferred that the simplified specific heat per unit volume, *ρ*″*c*″, is a constant. For the material thermal conductivity parameter after the equational simplification, it can be found that only one parameter of *k*_r_″ is inhomogeneous. The other two parameters of *k_θ_″* and *k*_0_ are uniform and have no singularity. Furthermore, there is a relationship, depicted as Equation (14), among the three parameters of *k*_r_″, *k*_θ_″, and *k*_0_. Thus, it provides conditions for realizing thermal cloaking.

(14) $$k_{r}^{\prime \prime }\cdot k_{\theta }^{\prime \prime }=k_{0}{}^{2}$$

There is a precondition for this simplification processing on material parameters, that is:

(15) $$\det(J_{xx^{\prime }})=\frac{r^{\prime }}{r-R_{1}}{\left(\frac{R_{2}-R_{1}}{R_{1}}\right)}^{2}\ll 1$$

However, this condition is often not satisfied for the outer boundary geometry of the annular thermal cloak meta-structure with a limited size, leading to the phenomenon that the external heat flow into the annular space will be distorted. That will result in a decrease in the thermal cloaking performance of the meta-structure unless the annular space is large enough. Nonetheless, inside this annular structure for thermal cloaking, that is, within the central circular area with a radius of *R*_1_ (Figure 1b), the primary thermal flow manipulation function still can confine the heat-flow transfer to the annular space without entering the central circular area. Therefore, simplifying the material parameters of the designed meta-structure is still an efficient mathematical processing, which can make the fabrication of the meta-structure easier.

### 2.3. Application of the Transformation Thermotics on Thermal Rotation Meta-Structure

Figure 1a,c depict the plane circular structure spaces before and after the coordinate transformation for designing a thermal rotation meta-structure. The circular spaces marked as *Ω* and *Ω*′ are the plane-limited spaces before and after the coordinate transformation, respectively. When the heat flow, *Q*, enters the coordinate-transformed space, *Ω*′, the direction of heat-flow transmission rotates by a certain angle of *θ*_0_ (Figure 1c). Meanwhile, the heat flow converges to the circular area with a radius of *R*_1_ (Figure 1c), so that the angle of *θ*_0_ rotates the heat-flow transmission direction in the central circular area.

Based on the coordinate transformation theory of transformation thermotics, for achieving the thermal rotation with the rotation angle of *θ*_0_, the main procedure of coordinate transformation from the *Ω* to the *Ω*′ is described as follows. No coordinate transformation is performed for the outer regions where *r* > *R*_2_. The following coordinate transformation is performed for the central circular area where *r* < *R*_1_.

(16) $$\left\{ \begin{array}{l} r^{\prime }=r\\ \theta^{\prime }=\theta +\theta_{0} \end{array}\right.$$

While for the annular region of the thermal rotation meta-structure where *R*_1_< *r* < *R*_2_, the following coordinate transformations are performed.

(17) $$\left\{ \begin{array}{l} r^{\prime }=r\\ \theta^{\prime }=\theta +\theta_{0}\frac{f(R_{2})-f(r)}{f(R_{2})-f(R_{1})} \end{array}\right.$$

where *f*(*r*) is a continuous function related to the *r*. The above coordinate transformation is to make the heat flow from the outside into the thermal rotation annular region (*R*_1_< *r* < *R*_2_) and then rotate so that when the heat flow enters the central region (*r* < *R*_1_), *θ*_0_ can rotate the heat-flow transfer direction concerning the heat-flow direction in the outer region (*r* > *R*_2_).

According to the above coordinate transformation mode, the transformation matrix of **T^−1^** is calculated as:

(18) $$T^{-1}=J_{{xx}^{\prime }}^{-1}J_{{xx}^{\prime }}^{-T}\det(J_{{xx}^{\prime }})=R(\theta {}^{\prime })J_{{\theta \theta }^{\prime }}^{-1}J_{{\theta \theta }^{\prime }}^{-T}R(-\theta {}^{\prime })T^{-1}=\left(\begin{array}{l} {\left(T^{-1}\right)}_{11}{\left(T^{-1}\right)}_{12}\\ {\left(T^{-1}\right)}_{21}{\left(T^{-1}\right)}_{22} \end{array}\right)$$

where

(19) $${\left(T^{-1}\right)}_{11}=1-2tcos(\theta {}^{\prime })sin(\theta {}^{\prime })+2tcos2(\theta {}^{\prime })$$

(20) $${\left(T^{-1}\right)}_{22}=1+2tcos(\theta {}^{\prime })sin(\theta^{\prime })+2tcos2(\theta {}^{\prime })$$

(21) $${\left(T^{-1}\right)}_{12}={\left(T^{-1}\right)}_{21}=2tcos(\theta {}^{\prime })-t(cos2(\theta {}^{\prime })-sin2(\theta {}^{\prime }))$$

(22) $$t=\theta_{0}rf{}^{\prime }(r)=r\theta_{0}/(R_{2}-R_{1})$$

Hence, when the thermal conductivity of the meta-structural background material is *k_b_*, the thermal conductivity of the thermal rotation meta-structure after the coordinate transformation can be obtained as:

(23) $$k{}^{\prime }=k_{b}\cdot T^{-1}$$

Based on the characteristics of the transformation matrix, the thermal conductivity of the thermal rotation meta-structure is related to the position and azimuth in this structure. Therefore, the thermal rotation meta-structure can exhibit the characteristics of thermal anisotropy and inhomogeneity through the above mathematical processing.

### 2.4. Application of the Transformation Thermotics on Thermal Concentration Meta-Structure

Figure 1a,d depict the plane circular structure spaces before and after the coordinate transformation for designing a thermal concentration meta-structure. Similar to the above two thermal meta-structures, the circular spaces marked as *Ω* and *Ω*′ are the plane-limited spaces before and after the coordinate transformation. After the coordinate transformation, the transmission path and direction of the heat flow changed after entering the space marked as *Ω*′, concentrating towards the central circular area with a radius of *R*_1_ (Figure 1d).

**Figure 2:**
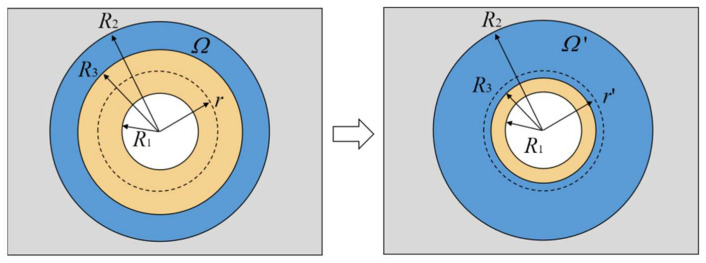
Schematic of an intermediate coordinate transformation process for the thermal concentration meta-structure.

Based on the transformation thermotics, for realizing the thermal concentration in Figure 1d, an intermediate procedure of coordinate transformation, as shown in Figure 2, is performed on the circular space marked as *Ω*′. In the radial direction, the coordinate transformation compresses the space where *r* < *R*_3_ to the space where *r* < *R*_1_, and stretches the space where *R*_3_ < *r* < *R*_2_ to the space where *R*_1_ < *r* < *R*_2_. While in the annular direction, the coordinate transformation does not stretch or compress the space. The compressed and stretched areas fill or compensate each other within the annular space, so the total space after and before transformation remains unchanged. After the coordinate transformation, the entire circular space is divided into two parts: the central area with a radius of *R*_1_, and the annular area where *R*_1_ < *r* < *R*_2_. When the heat flow passes through this annular region, the direction is changed from the horizontal transport direction to the direction concentrating towards the central region (*r* < *R*_1_). Thus, the compressed central region (*r* < *R*_1_) achieves the thermal manipulation for heat-flow concentration. Accordingly, the coordinate transformation mode is:

(24) $$\{\begin{array}{c} r{}^{\prime }=\frac{R_{1}}{R_{3}}r{}^{}(0\leq r\leq R_{3})\\ r{}^{\prime }=\frac{R_{2}-R_{1}}{R_{2}-R_{3}}r+\frac{R_{1}-R_{3}}{R_{2}-R_{3}}R_{2}{}^{}\;\;\;(R_{3}\leq r\leq R_{2})\\ \theta {}^{\prime }=\theta \end{array}$$

From this calculation, the transformation matrix of **T**, denoted as (*x*, *y*) → (*r*, *θ*) → (*r*′, *θ*′) → (*x*′, *y*′), is:

(25) $$T^{-1}=J_{xx{}^{\prime }}^{-1}J_{xx{}^{\prime }}^{-T}\det(J_{xx{}^{\prime }})=R(\theta {}^{\prime })J_{\theta \theta {}^{\prime }}^{-1}J_{\theta \theta {}^{\prime }}^{-T}R(-\theta {}^{\prime })$$

Then, according to Equation (3), the thermal conductivity parameters of a thermal concentration meta-structure after coordinate transformation can be calculated, namely:

(26) $$\{\begin{array}{c} k_{r^{\prime }}^{\prime }=k_{h},\;\;\;\;\;\;k_{\theta^{\prime }}^{\prime }=k_{h}\;\;\;\;\;(0\leq r{}^{\prime }\leq R_{3})\\ k_{r^{\prime }}^{\prime }=\frac{r{}^{\prime }+R_{2}\frac{R_{3}-R_{1}}{R_{2}-R_{3}}}{r{}^{\prime }}k_{h},\;\;\;\;k_{\theta^{\prime }}^{\prime }=\frac{r{}^{\prime }}{r{}^{\prime }+R_{2}\frac{R_{3}-R_{1}}{R_{2}-R_{3}}}\;\;k_{h}\;\;\;\;(R_{3}\leq r{}^{\prime }\leq R_{2}) \end{array}$$

where, *k_h_* is the thermal conductivity of the meta-structural background material, *k′_r′_* is the thermal conductivity in the radial direction of thermal concentration meta-structure after the coordinate transformation, and *k*′*_θ′_* is the thermal conductivity in the annular direction of thermal concentration meta-structure after the coordinate transformation.

It is inferred from Equation (26) that the thermal conductivity parameters of the thermal concentration meta-structure also exhibit inhomogeneity and anisotropy characteristics. Its radial-directional thermal conductivity, *k*′*_r_*_′_, is much larger than its annular-directional thermal conductivity, *k*′*_θ_*_′_. Meanwhile, the *k*′*_r_*_′_ and the *k*′*_θ_*_′_ have the following relationship: the product of the thermal conductivity values in the two directions is equal to the square of the background material thermal conductivity.

(27) $$k_{r^{\prime }}^{\prime }\cdot k_{\theta^{\prime }}^{\prime }=k_{h}{}^{{}_{2}}$$

Actually, no single material that can satisfy the thermal conductivity characteristics of Equation (27) has been found in nature. Therefore, in this case, according to the effective medium approximation theory [35,36,37], the thermal conductivity parameter designing requirements of the thermal concentration meta-structure can be approximately satisfied by reasonably employing the dissimilar materials with their structural and arrangement forms. The effective medium approximation theory provides efficient paths for realizing thermal metamaterials based on the transformation thermotics. The theory holds that using multiple homogeneous and isotropic natural materials, several different materials are reasonably combined and arranged according to specific structures designed to approximately fit the holistic metamaterial parameters with characteristics of anisotropy and inhomogeneity [35,36,37,38]. Summarily, a holistic thermal meta-structure can be equivalent to an orderly combination of some sub-structures composed of various single materials. The artificial design and adjustment of thermal metamaterials can be achieved by changing the physical properties, geometries, arrangements, chemical composition and other factors of the sub-structures and/or unit materials in the holistic thermal meta-structure.

## 3. Methods

### 3.1. Design and Numerical Simulation Analysis

According to the above-stated transformation thermotics, multiple meta-structural coordinate transformation modes, and effective medium approximation theory, metal Cu and PDMS were employed to construct modular geometries for various arrangements. Designing the configuration geometries and arrangement patterns of modules with Cu and PDMS is desired to effectively manipulate heat flow to achieve multiple functions, including thermal concentration, thermal cloak, thermal rotation, and thermal dispersion. These thermal functions can be converted using the thermal meta-structural devices composed of convertible arrangements of multiple identical modules.

The article will elaborate on the apparent design idea and procedures separately. During the design process, we employed commercial simulation software, COMSOL Multiphysics 5.4, to conduct thermal simulations to optimize the design.

We also use the software for numerical simulation analysis on the thermal features of the as-designed meta-structural devices. To further quantitatively analyze the thermal properties of the convertible thermal meta-structure devices, cross-transversal lines were selected on the numerically simulated thermal fields of the meta-structures for reading and plotting the relevant temperature and heat flux curves along those transversal lines.

### 3.2. Hybrid Manufacturing via SLA 3D Printing and Surface Metallization

The convertible thermal meta-structure device designed and fabricated in this work was a planar structure. A professional SLA 3D printer (Formlabs Form3) was employed to print resin modules to conduct surface metallization on them and then compose meta-structural devices. The *Z*-axis layer-by-layer thickness accuracy and the laser spot diameter of the SLA printer were 25 μm and 85 μm, manifesting the relatively high precision of the SLA printer to guarantee high geometrical accuracy and good surface roughness of the as-printed resin parts. The raw material to be printed by SLA was liquid photosensitive resin. After laser-induced curing, the resin exhibited high stiffness and low creep and was suitable for conceptual modelling and functional prototyping. A surface metallization technical route of electroless plating hybrid with electroplating was tailored to prepare a metal Cu layer on the surface of SLA-printed resin structures. We called the fabrication method of SLA combined with the hybrid plating “hybrid manufacturing” for Cu-contained thermal meta-structural devices. The main procedures of “hybrid manufacturing” can be briefly described as follows.

1. The 3D models of modules to compose several thermal meta-structural devices were imported to the SLA printing process software system and then printed using a 25 μm layer thickness and a 250 mW laser power. The as-printed solid parts were cleaned using alcohol to remove the uncured liquid resin from their surface.
2. The as-printed and air-dried resin modules were electrolessly plated to prepare a Cu-containing conductive layer on the surface as an intermediate and conductive layer for subsequent electroplating since the as-printed solid resin was not conducive. Silicon–oxygen coupling agent acetone solution was used for the initial surface activation of the as-printed solid resin, aiming to enhance the adhesion of Cu ions on the resin surface. Then, the parts were soaked in the electroless plating solution, which contained CuSO_4_, HCHO, C_4_O_0_H_4_KNa, and NaOH, for Cu ions attaching to the resin surface and undergoing a reduction reaction at a set temperature of 30°, according to a non-catalytic reaction, causing Cu_2_O particles to be reduced: 2Cu^2+^ + HCHO + 5OH^−^ → 2HCOO^−^ + 3H_2_O + Cu_2_O. Then, the Cu_2_O was further reduced to particulate copper, according to a reaction: Cu_2_O + 2HCHO + 2OH^−^ → 2Cu + 2HCOO^−^ + H_2_ + H_2_O. The duration of the electroless plating of Cu on the resin modules was 30 min.
3. Next, Cu electroplating was performed on the module parts containing the electroless-plated conductive layer of Cu to obtain a thicker and denser metal Cu thermal conductive layer on their surface. The main reagent components of the professional Cu electroplating solution, including NaKC_4_H_4_O_6_, K_2_HPO_4_, and K_6_[Cu(P_2_O_7_)_2_], was employed in a lab-used electrolyzer. An electroplating instrument, including DC stabilized power supply, a Harlem tank, and the heater was utilized for plating Cu at room temperature. The pH value of the plating solution was tailored as 7. The current density for electroplating was determined as 0.5 A/dm^2^. The duration of the Cu electroplating was 45 min.
4. After the surface plating of Cu was conducted, a PDMS liquid was configured and poured into the grooves of the modules. The PDMS played the role of heat insulation and packaging. Subsequently, the module parts with liquid PDMS were placed in a room-temperature environment for 24 h to cure.
5. The multiple modules with a solid resin, Cu surface layer, and cured solid PDMS were arranged in different patterns to assemble and compose meta-structural devices with several thermal-flow manipulative forms, including thermal cloak, thermal rotation, thermal concentration, and thermal dispersion.

### 3.3. Thermal Testing Verification

Heat-flow manipulation verification tests were conducted on the fabricated thermal meta-structural devices using a platform consisting of an infrared thermal imager, a high-temperature water (90 °C) bath, and a low-temperature water bath (room-temperature of ~20 °C). Thus, the temperature difference was built between the two opposite sides of the thermal meta-structural device, which was imaged using the high-resolution infrared thermal imager (Compact Pro model from Seek Thermal Inc., Calle Real, Goleta, CA, USA) with a test temperature range of −40~330 °C. In this work, as a demonstration, we obtained the temperature nephograms of thermal cloak and thermal concentration meta-structural devices during the testing process, respectively.

## 4. Convertible Thermal Meta-Structural Design

**Figure 3:**
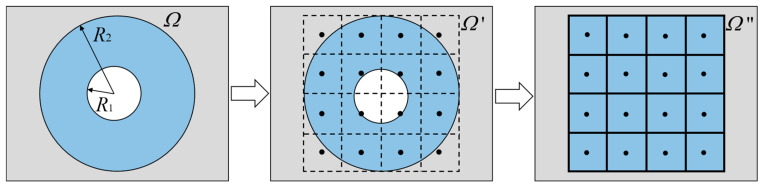
Schematic discretization of an annular meta-structural plane into sixteen square units.

The annular region in the planar structure was discretized into sixteen square units as the modules. These square units were used to approximately replace the annular structure area, as shown in Figure 3. The arrangement of the square units was similar to a “jigsaw puzzle”. Each unit could be rotated at a certain angle. Therefore, the convertible thermal manipulation functions of the meta-structure could be achieved by rotating the units at specified angles and arraying them according to different alignment strategies. To this end, we should determine the heat-flow transfer characteristics and paths in the entire convertible meta-structure and then design the heat-flow direction or deflection angle within each square unit. By designing the structural geometric characteristics of each square unit, the geometric parameters of each unit were tailored.

**Figure 4:**
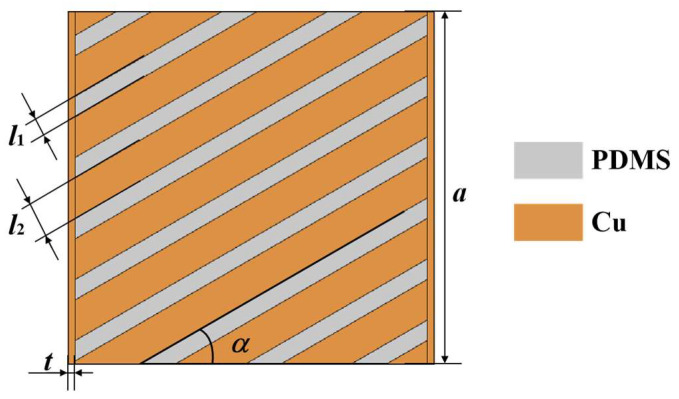
Schematic of one square unit as a module in the meta-structure.

This work introduced a grid geometry form into the square units, as shown in Figure 4. We used PDMS material as one strip element with a width denoted as *l*_1_, and copper metal as the other strip element with a width denoted as *l*_2_. The stripe-shaped elements of the two materials were staggered and merged into a square unit with a side length denoted as *a*. The stripe-shaped elements of the square unit had an included angle, denoted as *α*, with the horizontal direction. To improve the heat transfer efficiency, the copper material edge strips with a width denoted as *t* were designed on both sides of the square unit. Considering the enhancement in heat transfer efficiency and the feasibility of subsequent fabrication and thermal tests, we determined the geometric parameters: *a* = 10 mm, *l*_1_ = 0.5 mm, *l*_2_ = 1 mm, and *t* = 0.2 mm.

**Figure 5:**
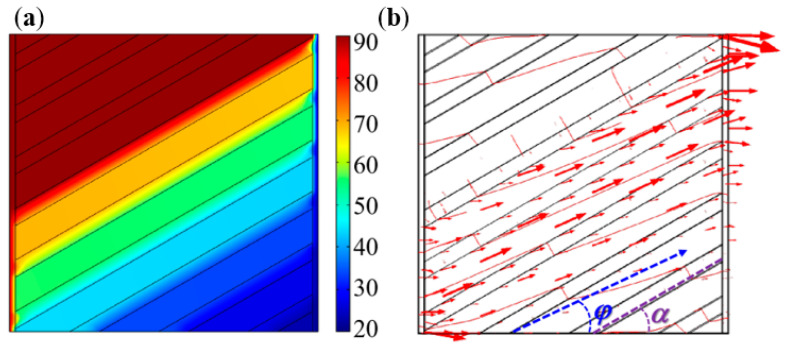
Thermal simulation results for a square unit with *α* 30°: (**a**) nephotogram of temperature fields on the surface; (**b**) heat flow direction with an inclined angle *φ*.

The design theory of the square unit was an approximate design idea based on the effective medium theory. There was still a certain deviation between the actual heat-flow transfer direction and the stripe-shaped element extension direction in the same square unit. For instance, if the angle, *α*, of the stripe-shaped elements was set to 30°, the thermal simulation results of the square unit are shown in Figure 5. The thermal simulation boundary conditions included the ambient temperature of room-temperature (20 °C), the left end boundary temperature of 90 °C, the right end boundary temperature of 20 °C, and thermal insulation of the upper and lower surfaces of the square unit.

Figure 5 demonstrated that the inclined angle of the heat-flow direction, *φ*, did not coincide with the inclined angle of the stripe-shaped elements *α*. In other words, the square unit’s heat transfer exhibited a certain deflection phenomenon. The calculated heat-flow direction angle, *φ*, was 27.2°, which had only a 9.3 % error with *α*, when the *α* was determined as 30°. We also calculated the *φ* values when *α* was 45°, 60°, and 75°, respectively. The simulation calculated results indicated that the two angles were still approximately equal under the above conditions. Therefore, the square unit could be approximately considered to make the heat-flow transfer directions according to the designed geometry orientations of the stripe-shaped elements in the square units.

**Figure 6:**
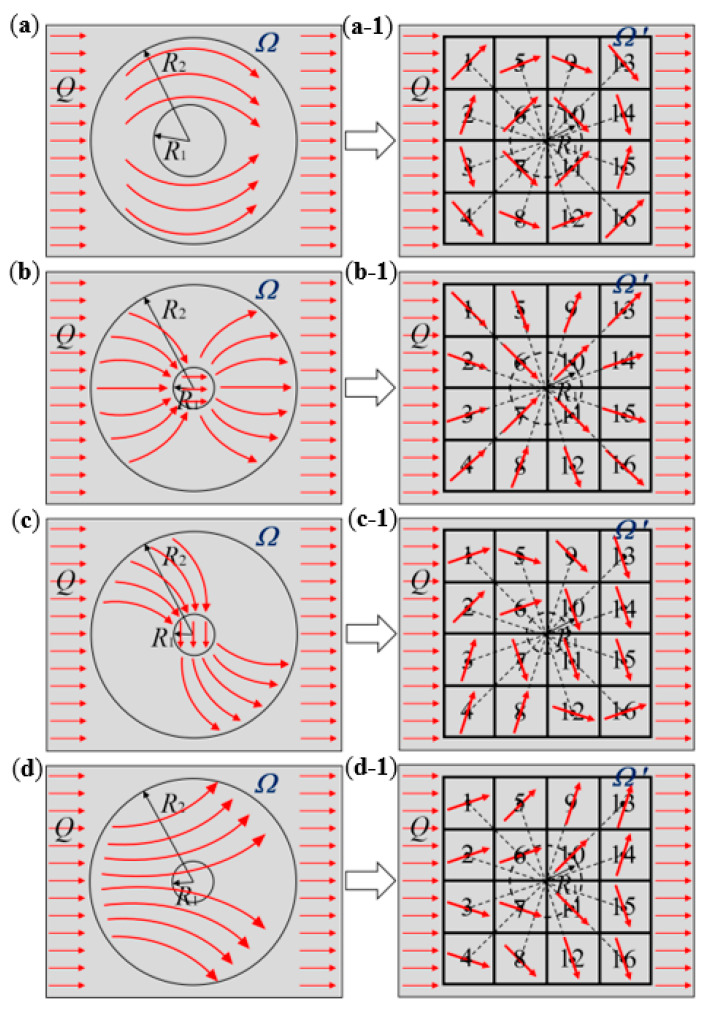
Schematic diagrams of heat-flow transfer directions in the meta-structures of thermal cloak (**a**,**a-1**), thermal concentration (**b**,**b-1**), thermal rotation (**c**,**c-1**), and thermal dispersion (**d**,**d-1**), before (**a**–**d**) and after (**a-1**–**d-1**) spatial discretization to sixteen square unit modules.

**Figure 7:**
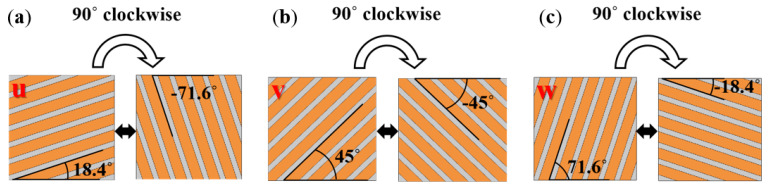
The square unit modules, with 18.4° (**a**), 45° (**b**), and 71.6° (**c**) angled stripe-shaped elements, constitute the convertible thermal meta-structural devices.

**Figure 8:**
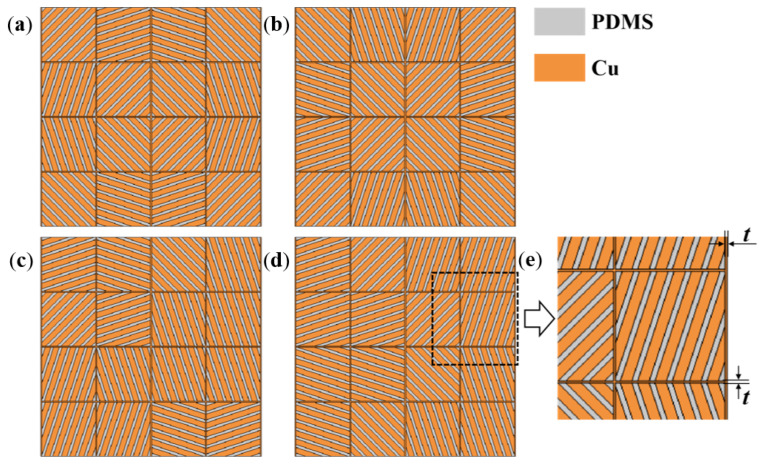
Sketches of the thermal meta-structural devices constituted by three types of square unit modules, for the thermal manipulation functions of thermal cloak (**a**), thermal concentration (**b**), thermal rotation (**c**), and thermal dispersion (**d**), with a partially enlarged schematic diagram (**e**).

The effectiveness of the meta-structural thermal manipulation functions based on the above-mentioned “jigsaw puzzle” design idea also largely depended on the number of square unit modules per unit area of the meta-structural plane. That is similar to the relationship between the picture and the pixel. That is, the more pixels that make up a picture, the clearer the picture. Theoretically, the more square units in a convertible meta-structure per unit area, the better the thermal manipulation performance. Herein we selected the number of the square unit modules to compose one convertible meta-structure as 16, i.e., 4 × 4, for the design, simulation analyses and verification. As exhibited in Figure 6 and Table 1, through the following different arrangements of the modules, multiple thermal manipulations can be achieved based on the different discrete heat-flow straight directions within the modules. Accordingly, a total of six kinds of square unit modules, with the heat-flow transfer angles of 18.4°, 45°, 71.6°, −18.4°, −45°, and −71.6°, were used for the meta-structures with multiple functions including thermal concentration, thermal cloak, thermal rotation, and thermal dispersion. The orientations of the stripe-shaped elements in the square units roughly equated to the *φ* values. While the stripe-shaped elements with the inclined angles of −18.4°, −45°, and −71.6° could be acquired by clockwise or counterclockwise rotating 90° of the square units with 71.6°, 45°, and 18.4° angled stripe-shaped elements, respectively. Therefore, the types of the square unit modules constituting the convertible thermal meta-structural devices were simplified into three types: the square units with 18.4°, 45°, and 71.6° angled stripe-shaped elements, respectively shown in Figure 7 and Figure 8.

## 5. Results and Discussion

### 5.1. Thermal Simulation Analysis of the Meta-Structures

**Figure 9:**
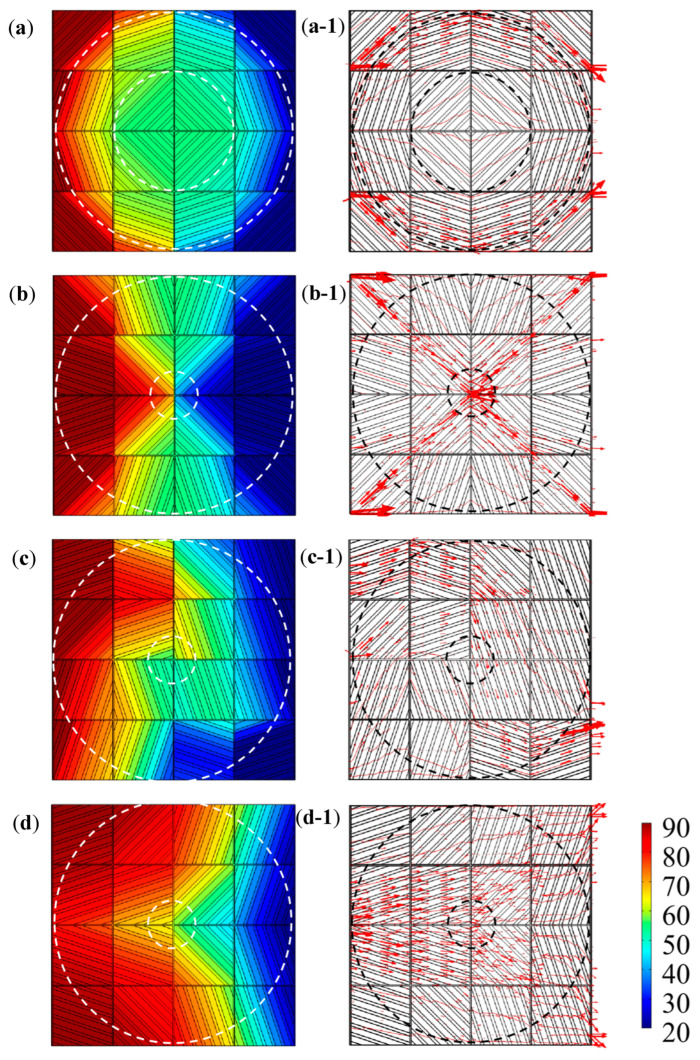
The simulation results of temperature fields (**a**–**d**) and heat-flow transfer directions (**a-1**–**d-1**) in convertible meta-structures for the thermal manipulations of thermal cloak (**a**,**a-1**), thermal concentration (**b**,**b-1**), thermal rotation (**c**,**c-1**), and thermal dispersion (**d**,**d-1**).

**Figure 10:**
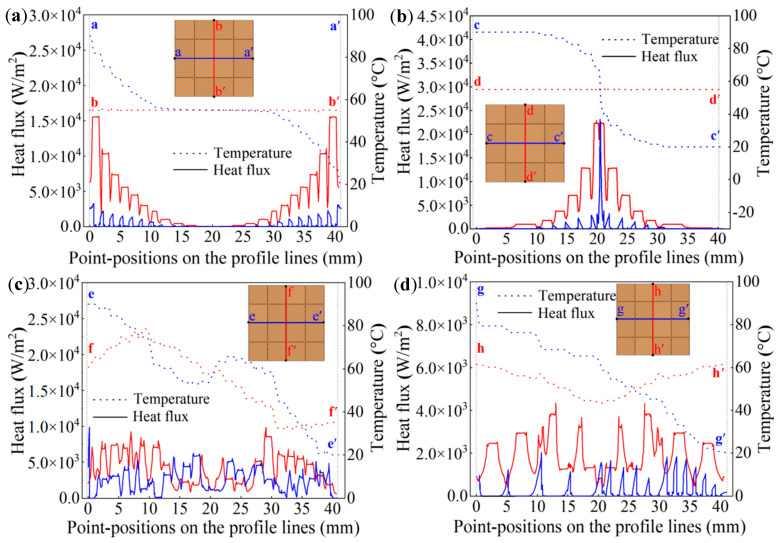
The temperature change curves (dotted lines) and heat flux change curves (solid lines) along selected cross-sectional lines within the thermal cloak (**a**), thermal concentration (**b**), thermal rotation (**c**), and thermal dispersion (**d**) meta-structures. Note: The blue lines are the changes in temperature and heat flux values along the illustrated transverse sectional lines marked as a-a’ (**a**), c-c’ (**b**), e-e’ (**c**), and g-g’ (**d**). The red lines are the changes in temperature and heat flux values along the illustrated longitudinal sectional lines marked as b-b’ (**a**), d-d’ (**b**), f-f’ (**c**), and h-h’ (**d**).

Under the thermal simulation boundary conditions, including an ambient temperature of room-temperature (20 °C), the left end boundary temperature of 90 °C, the right end boundary temperature of 20 °C, and thermal insulation of the upper and lower surfaces of the meta-structure, Figure 9 and Figure 10 give the simulation results of temperature fields, heat-flow transfer directions, temperature and heat flux change curves in the convertible meta-structures for the thermal manipulations of the thermal cloak, thermal concentration, thermal rotation, and thermal dispersion.

In the thermal cloak meta-structure, the thermal flow transferred around the central cloaking area, and the temperature field remained almost unchanged (Figure 9a,a-1). In contrast, the thermal flow was directed to the center of the thermal concentration meta-structure, thereby increasing the temperature in the central area (Figure 9b,b-1). While the direction of heat transfer in the thermal rotation meta-structure was rotated, the horizontal rightward heat flow transfer was reversed downward in the meta-structure. Thus, the temperature at the upper end of the rotation meta-structure was higher than that at the lower end (Figure 9c,c-1). In the thermal dispersion meta-structure, the horizontally transported heat flow was gradually diffused in the directions of the upper and lower ends, respectively, so that the temperature in the middle region was significantly lower. The convertible thermal meta-structural simulation results were consistent with the initial design requirements. The overall meta-structure exhibited thermal characteristics according to human will through different thermal manipulation modes by the arrangement and combination of the three types of modules.

To further quantitatively analyze the thermal properties of the convertible thermal meta-structures, sectional lines by truncation in the meta-structures were selected along the central axes to plot the relevant change curves of the temperature and heat flux, as indicated in Figure 10.

In the middle part of the vertical section lines marked as a-a’ and b-b’ in the thermal cloak meta-structure, the temperature and heat flux curves exhibited horizontal lines almost without fluctuation. The temperature and the heat flux were stable at 55 °C and in the range of 50~100 W/m^2^, respectively. That was, in the central area of the meta-structure, the temperature value remained constant. The heat flux value was also stabilized to a minimal value in the meta-structural central area, so the structure exhibited thermal cloaking characteristics (Figure 10a).

In the horizontal direction of the thermal concentration structure, the temperature change curve along the vertical section line marked as c-d’ showed a sharp drop in the middle section. A relatively large temperature gradient was generated in the central area of the structure. The heat flux curves in the horizontal and vertical directions exhibited similar curve shapes, so the heat flux value in the middle section of the curve increased evidently. Consequently, a pronounced thermal concentration characteristic was created in the central region of the meta-structure (Figure 10b).

Two section lines marked as e-e’ and f-f’ in the horizontal and vertical directions of the thermal rotation meta-structure were traced, respectively. It was indicated that the fluctuation of the heat flux change curve in the horizontal direction was relatively stable within a certain range. In comparison, the heat flux change in the vertical direction was larger in the beginning and end sections. It indicated that the heat flux increased significantly in the vertical direction, which in turn showed the direction change of the heat flow from the horizontal direction to the vertical direction. For the horizontal temperature change curves, the temperatures in the beginning section were much lower than those in the end section, showing a temperature inversion phenomenon (Figure 10c).

In the thermal dispersion meta-structure, the fluctuation of the heat flux change curve in the horizontal direction was relatively stable. The heat flux curve in the vertical direction showed that the heat flux in the upper and lower parts of the structure was more prominent, but smaller in the middle part. Thus, in the temperature change curve along the vertical direction, the temperatures at both ends were higher than those in the middle, showing a thermal dispersion characteristic (Figure 10d).

Although the numerical simulations, according to idealized modelling, the numerical simulations could demonstrate the thermal manipulation features of the designed thermal meta-structures. However, due to various limitations, the numerical simulations could not be adequately represented for practically fabricated meta-structural devices. Nevertheless, through the experimental testing verification on the as-fabricated devices, the temperature variation trend of the essential thermal manipulation process could also be reflected.

### 5.2. Fabrication and Thermal Testing Results of the Meta-Structures

**Figure 11:**
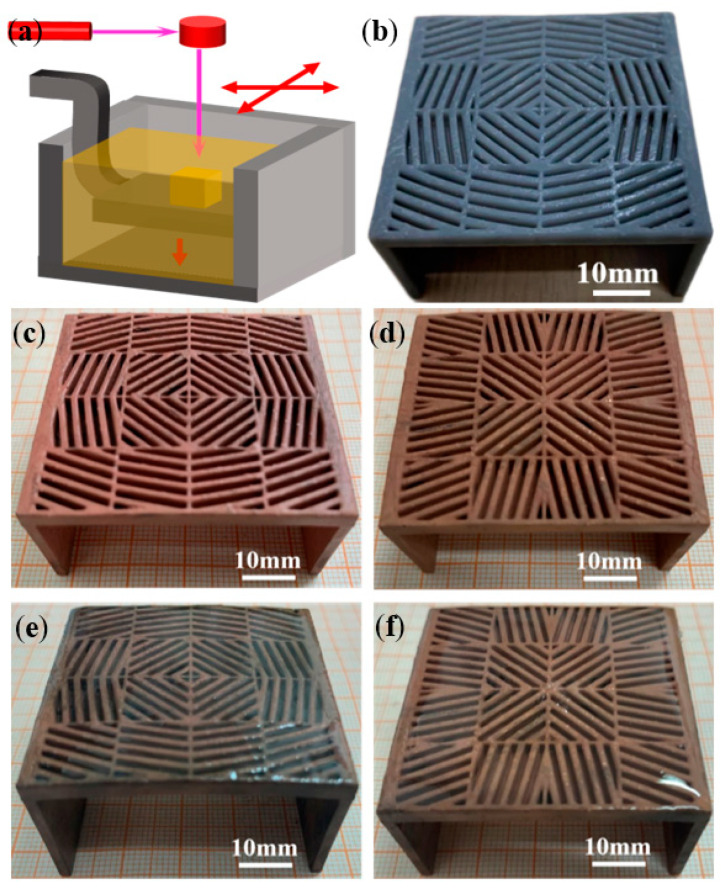
Fabrication results of the meta-structural devices: schematic of SLA (**a**) for 3D printing a solid resin material meta-structural device (**b**); the macroscopic appearance of thermal cloak (**c**) and thermal concentration (**d**) meta-structural devices after surface copper metallization; the macroscopic appearance of thermal cloak (**e**) and thermal concentration (**f**) meta-structural devices after nearly adiabatic packaging using PDMS.

Figure 11 shows the macroscopic appearances of thermal cloak and concentration meta-structural devices after SLA 3D printing (Figure 11a,b), surface metallization (Figure 11c,d), and nearly adiabatic packaging using PDMS (Figure 11e,f). The SLA 3D printing method guaranteed a high geometric accuracy of the square unit modules. The surface metal layers obtained by the hybrid plating all covered the surface of the entire meta-structures relatively uniformly. The cured PDMS for encapsulation ensured a certain degree of thermal insulation upon the upper and lower surfaces of the meta-structural devices and the interstices between the stripe-shaped elements with a Cu surface layer.

**Figure 12:**
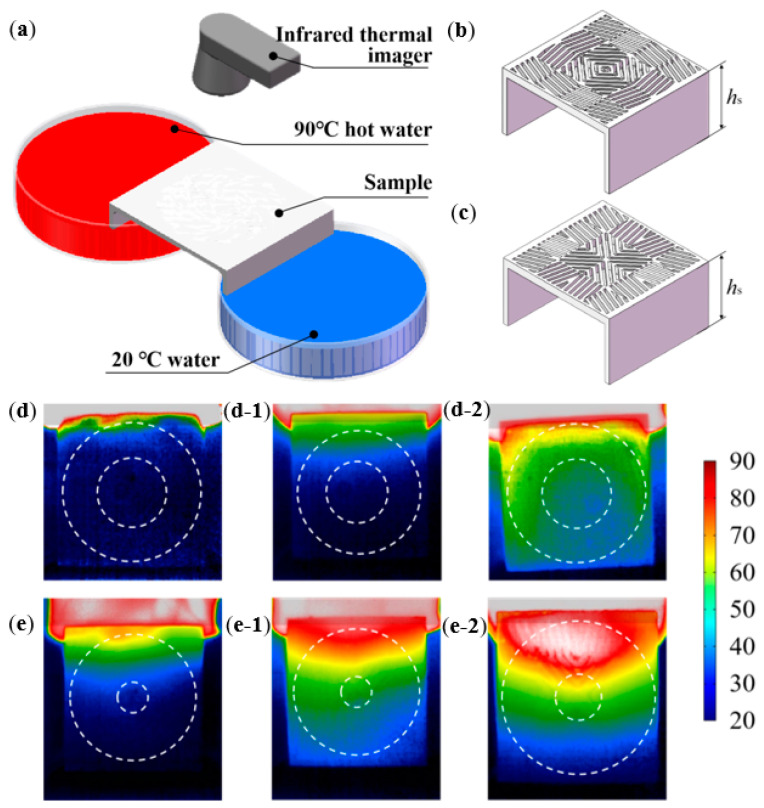
Experimentally measurement (**a**) of the temperature field nephograms of the thermal cloak (**b**) and concentration (**c**) meta-structural devices via the hybrid manufacturing: the temperature field nephograms after measuring for 10 s (**d**,**e**), 60 s (**d-1**,**e-1**), and 180 s (**d-2**,**e-2**) on the thermal cloak (**d**–**d-2**) and concentration (**e**–**e-2**) meta-structures.

Figure 12a sketches the experimental measurement devices for monitoring the temperature changes on the meta-structural devices. To facilitate heat transfer and thermal testing, two vertical plates with a height, denoted as *h_s_* (Figure 12b,c), of 25 mm were printed at both ends of the prepared structure. At the beginning of the thermal test, the heat of 90 °C hot water was gradually transferred from the vertical plate to the plane plate with square unit modules. As the heat flew into the thermal cloak (Figure 12d–d-2) and concentration (Figure 12e–e-2) meta-structural devices gradually increased, the heat flow achieved a steady state when the testing duration went on for 180 s. After the heat-flow transfer was stabilized, the temperature nephogram on the thermal cloak or concentration meta-structures had a high consistency with that simulated by the computer. Thus, the thermal test results of the two meta-structures showed the expected thermal features, verifying the design theory and experimental methods.

### 5.3. Engineering Advantages of Hybrid-Fabricated Convertible Thermal Meta-Structures

So-called “convertible” in the designed and fabricated meta-structures depends on the changes in the arrangements and ordered rotations of the square unit modules with the spacer geometry of stripe-shaped dissimilar materials of thermally conductive metal Cu and nearly insulating PDMS. Although the copper geometry can also be directly formed by other 3D printing methods such as SLM and SLS, the surface roughness and the geometric accuracy of the as-printed structures are commonly challenging to meet the meta-structural design requirements. The SLA 3D printing, with the assistance of a hybrid plating method, for forming a high thermal conductivity layer by metallization on the solid resin surface, can ensure high geometric accuracy, a relatively low cost, and a pronounced convenience of mass manufacturing for the thermal meta-structural devices. The so-called “convertible” is accessible and relatively easy to operate in the potential applications. Although it seems that so many unit modules are included in a single meta-structure, only a few units are required to be rotated and arranged to be assembled into various thermal meta-structures. This design idea has advantages in terms of manufacturing cost, ease of application, repairability and replaceability, and flexible applicability.

However, it must be noted that the functional realization of the convertible thermal meta-structural devices also depends on the geometry size effect and the appropriate choice of materials to compose the devices. In general, the smaller the size of the complex geometric structure in the meta-structural devices, the better the heat flow features that agree with the numerical simulation of the idealized model. In addition, completely insulating materials are challenging to obtain in reality. In this work, the thermal insulation effect of PDMS was still limited, although in sharp contrast to the copper with high thermal conductivity. A complete local structural thermal insulation guarantee ensures the continuity and long-term effectiveness of the thermal manipulation function.

Nonetheless, the design and verification of convertible thermal meta-structures in this work and the proposed hybrid fabrication method demonstrate the potential applications of convertible thermal meta-structural. Moreover, the preparation and verification methods of thermal meta-materials will be enriched. The design and research on more complex thermal meta-materials can be effectively promoted.

## 6. Conclusions

According to the transformation thermotics, the convertible thermal meta-structural devices were designed by discretizing a two-dimensional plane and separating multiple square unit modules with stripe-shaped dissimilar materials. The meta-structures were fabricated using SLA 3D printing and surface metallization by electroless plating and then electroplating. The thermal manipulation characteristics were preliminarily verified. Some conclusions can be summarized as follows.

(1) Using multiple dispersed square unit modules to replace the annular region of the thermal meta-structure exhibited different thermal flow functions, including thermal cloak, concentration, rotation, and dispersion, through the unique geometric design of the heat-flow transfer direction on each module. By rotating each square unit module at a specific angle and arranging the modules, the convertibility among different thermal manipulation functions was achieved.
(2) Thermal simulations and plotted temperature and heat flux change curves of the thermal meta-structures indicated that the convertible thermal meta-structure had a solid ability to change the directions of heat flows. It was also verified in the experimental thermal tests of artificially fabricated thermal cloak and concentration meta-structural devices.
(3) The convertible thermal meta-structure devices with good geometric accuracy were successfully fabricated via a hybrid manufacturing path of “SLA 3D printing—electroless plating of Cu—electroplating of Cu—PDMS packaging”. A uniform copper layer with a high thermal conductivity was achieved on the SLA-printed solid resin part to act as heat conduction elements in the modules. The proposed hybrid manufacturing method can exhibit a high geometric accuracy, a relatively low cost, and a pronounced convenience of mass manufacturing for thermal meta-structural devices.

## Figures and Tables

**Table 1:** The heat-flow transfer inclined angles, denoted as *φ*, of the square unit modules in the thermal cloak (TClM), thermal concentration (TCoM), thermal rotation (TRoM), and thermal dispersion (TDiM) meta-structures sketched in Figure 6.

Module No. in TClM.	Module *φ* in TClM	Module No. in TCoM	Module *φ* in TCoM	Module No. in TRoM	Module *φ* in TRoM	Module No. in TDiM	Module *φ* in TDiM
No. 1	45°	No. 1	−45°	No. 1	18.4°	No. 1	18.4°
No. 2	71.6°	No. 2	−18.4°	No. 2	45°	No. 2	18.4°
No. 3	−71.6°	No. 3	18.4°	No. 3	71.6°	No. 3	−18.4°
No. 4	−45°	No. 4	45°	No. 4	71.6°	No. 4	−18.4°
No. 5	18.4°	No. 5	−71.6°	No. 5	−18.4°	No. 5	45°
No. 6	45°	No. 6	−45°	No. 6	18.4°	No. 6	18.4°
No. 7	−45°	No. 7	45°	No. 7	−71.6°	No. 7	−18.4°
No. 8	−18.4°	No. 8	71.6°	No. 8	71.6°	No. 8	−45°
No. 9	−18.4°	No. 9	71.6°	No. 9	−45°	No. 9	71.6°
No. 10	−45°	No. 10	45°	No. 10	−71.6°	No. 10	45°
No. 11	45°	No. 11	−45°	No. 11	−71.6°	No. 11	−45°
No. 12	18.4°	No. 12	−71.6°	No. 12	−18.4°	No. 12	−71.6°
No. 13	−45°	No. 13	45°	No. 13	−71.6°	No. 13	71.6°
No. 14	−71.6°	No. 14	18.4°	No. 14	−71.6°	No. 14	71.6°
No. 15	71.6°	No. 15	−18.4°	No. 15	−71.6°	No. 15	−71.6°
No. 16	45°	No. 16	−45°	No. 16	18.4°	No. 16	−71.6°

## Data Availability

Data available on request.
